# A Phase III Randomized Study Comparing a Chemotherapy with Cisplatin and Etoposide to a Etoposide Regimen without Cisplatin for Patients with Extensive Small-Cell Lung Cancer

**DOI:** 10.3389/fonc.2017.00217

**Published:** 2017-09-19

**Authors:** Thierry Berghmans, Arnaud Scherpereel, Anne-Pascale Meert, Vicente Giner, Jacques Lecomte, Jean-Jacques Lafitte, Nathalie Leclercq, Marianne Paesmans, Jean-Paul Sculier, for the European Lung Cancer Working Party (ELCWP)

**Affiliations:** ^1^Department of Intensive Care, Institut Jules Bordet, Centre des tumeurs, Université Libre de Bruxelles, Bruxelles, Belgium; ^2^Department of Oncological Emergencies, Institut Jules Bordet, Centre des tumeurs, Université Libre de Bruxelles, Bruxelles, Belgium; ^3^Department of Thoracic Oncology, Institut Jules Bordet, Centre des tumeurs, Université Libre de Bruxelles, Bruxelles, Belgium; ^4^Pneumologie et Oncologie Thoracique, CHU de Lille, Université de Lille, Lille, France; ^5^Hospital de Sagunto, Valencia, Spain; ^6^Hôpital Civil de Charleroi, Charleroi, Belgium; ^7^Data Centre, Institut Jules Bordet, Centre des tumeurs, Université Libre de Bruxelles, Bruxelles, Belgium

**Keywords:** small-cell lung cancer, chemotherapy, cisplatin, etoposide, extensive disease

## Abstract

**Introduction:**

In a literature meta-analysis, we showed survival benefits for regimens including cisplatin [hazard ratio (HR) 0.61; 95% confidence interval (CI), 0.57–0.66] and for those including etoposide (HR 0.65; 0.61–0.69). That benefit was mainly observed when etoposide alone or in combination with cisplatin was included in the chemotherapy regimens. Our objective was to determine if chemotherapy with both drugs improves survival in comparison to a non-platinum regimen with etoposide.

**Methods:**

Extensive small-cell lung cancer patients were randomized between cisplatin–etoposide (CE) and ifosfamide + etoposide + epirubicin regimen (IVE) between 2000 and 2013.

**Results:**

176 and 170 eligible patients were allocated to CE and IVE (315 deaths were required before analysis), respectively. Objective response rates were not significantly different: 60% with CE and 59% with IVE. No statistically significant difference in median survival and 1-year and 2-year was observed with rates of 9.6 months, 31 and 5% for CE and 10 months, 39 and 9% for IVE, respectively. HR was 0.84 (95% CI 0.68–1.05, *p* = 0.16). Only two prognostic factors for survival were retained in multivariate analysis: sex with HR = 0.69 (95% CI 0.49–0.97, *p* = 0.03) and performance status with HR = 0.53 (95% CI 0.49–0.97, *p* < 0.0001). After adjustment for these prognostic factors, HR for survival was 0.83 (95% CI 0.65–1.08, *p* = 0.17). There was more thrombopenia in the CE regimen and more leukopenia with IVE.

**Conclusion:**

Combination of CE failed to improve survival in comparison to an etoposide-containing regimen without cisplatin.

**Clinical Trial Registration:**

https://clinicaltrials.gov/ct2/show/NCT00658580?term=ELCWP+01994&rank=1, identifier NCT00658580.

## Introduction

Introduction of chemotherapy in the treatment of small-cell lung cancer (SCLC) modified significantly the prognosis of those patients, not only by achieving impressive objective response rates but also by impacting deeply on overall survival. Despite these achievements, few patients will be cured with a 5-year overall survival below 5% (1). Higher cure rates are obtained in patients with limited disease (LD) compared to those with extensive or metastatic disease (ED), LD being a strong prognostic factor (2).

Standard treatment for extensive disease, a disease where lesions cannot be encompassed in a single radiotherapy field, consists in combination chemotherapy. There remains discussion about the most active chemotherapy regimen. In the late 1990s when designing this trial, Americans mainly recommended “cisplatin (CDDP) and etoposide (VP16)” based-regimens and Europeans “cyclophosphamide–adriamycin–etoposide” based-combinations. Each regimen showed a differential toxicity profile with increased renal and neurological impairment in cisplatin-containing regimens and more cardiotoxicity with anthracycline derivatives.

As there was no valuable randomized trial comparing these so-called standard regimens, our cooperative group, the European Lung Cancer Working Party (ELCWP), performed a meta-analysis of the literature on the topic (3). 36 published randomized trials during the period 1980–1998 comparing first-line regimens were identified. They were grouped according to the regimen comparisons: CDDP-based regimen versus chemotherapy without CDDP or VP16 (group 1; 1 trial), VP16-based regimen without CDDP versus chemotherapy without VP16 (group 2; 17 trials), CDDP + VP16 versus a regimen without both drugs (group 3; 9 trials), and CDDP + VP16 versus a VP16 only regimen (group 4; 9 trials). Combined hazard ratios (HRs) were as follows: 0.70 [95% confidence interval (CI) 0.41–1.21] for group I, 0.73 (95% CI 0.67–0.78) for group II, 0.57 (95% CI 0.51–0.64) for group III, and 0.74 (95% CI 0.66–0.83) for group IV. Those data showed better effectiveness for the combination of cisplatin and etoposide, while improved survival was also observed separately for CDDP (HR = 0.61, 95% CI: 0.57–0.66) and for VP16 (HR = 0.65, 95% CI: 0.61–0.69).

Robustness of the results of that meta-analysis had to be confirmed by randomized trials. The purpose of the present trial was thus an attempt to confirm in terms of overall survival the superiority of the cisplatin + etoposide (CE) combination over an etoposide-containing regimen without cisplatin as often used in Europe. We have chosen the CE regimen as used in LD (4) and the IVE regimen with ifosfamide, epirubicin, and etoposide as shown in our prior trials (5). The ELCWP has shown that epirubicin is associated with better cardiac tolerance than adriamycin when combined to ifosfamide and etoposide (6).

## Patients and Methods

### Eligibility Criteria

To be eligible, patients with pathologically proven SCLC [using World Health Organization (WHO) classification] had to present with ED, defined as a disease with distant metastases or as a locoregional disease that could not be locally treated within a single radiotherapy field. Other inclusion criteria were as follows: no prior therapy (radiotherapy, chemotherapy, or surgery), Karnofsky performance status (PS) of at least 60, presence of an assessable lesion, no history of prior malignant tumor except non-melanoma skin cancer or *in situ* carcinoma of the cervix or a cured cancer (defined as a disease-free interval > 5 years), adequate hematological (WBC count ≥ 4,000 mm^3^ and platelet count ≥ 100,000 mm^3^), renal (serum creatinine < 1.3 mg/dl) and hepatic (serum bilirubin < 1.5 mg/dl) functions, no recent myocardial infarction (<3 months before date of diagnosis), no congestive cardiac failure and cardiac arrhythmia requiring medical treatment, no uncontrolled infectious disease, and no other serious medical or psychological factors that might prevent adherence to the treatment schedule. Patients with brain metastases can be included provided that they satisfied to the other inclusion criteria. Patients had to be accessible for follow-up and to provide informed consent. Study protocol was approved by the ethical committee of each participating institution.

### Therapeutic Schedule

Eligible patients were randomized between the CE regimen [cisplatin (90 mg/m^2^ d1) plus etoposide (100 mg/m^2^ d1 to 3)] and the ifosfamide + etoposide + epirubicin regimen [IVE: epirubicin (60 mg/m^2^ d1) plus etoposide (100 mg/m^2^ d1 to 3) plus ifosfamide (1.5 g/m^2^ d1 to 3)]. All drugs were intravenously (iv) administered. Cisplatin was given over 30 min in 250 ml NaCl 3%, after prehydration with 1,000 ml of 5% dextrose in 0.45% NaCl for 4 h and followed by a mannitol induced diuresis (12.5 g of mannitol injected as an iv bolus immediately prior to cisplatin administration and then as a continuous 20% solution 60 ml/h for the next 6 h) and a posthydration with 4,000 ml of 5% dextrose in 0.45% NaCl with 1.5 g KCl/l for the next 24 h. Diuresis and emesis had to be measured every 6 up to 24 h thereafter and if urine output decreased to <75 ml/h, furosemide (40 mg) had to be administered iv. Etoposide was diluted in 250 ml NaCl 0.9% and infused over 1 h, just after cisplatin administration. Epirubicin was given as a short infusion before etoposide. Ifosfamide was diluted in 1 l NaCl 0.9% and administered iv over 3 h. Mesna was infused at a dose of 300 mg/m^2^ just before ifosfamide and then every 4 for 72 h.

Cycles were repeated every 3 weeks, with response evaluation after the three first ones. In case of no response, patients went off treatment. In case of response, chemotherapy was administered until complete response or unacceptable toxicity or best response, defined as non-improved response by three further courses of chemotherapy. Response had to be assessed every three cycles. At relapse, if the delay since the last chemotherapy cycle was more than 6 months, the same chemotherapy regimen as initially was given again. Otherwise, patients were off trial. Prophylactic cranial irradiation was not mandatory in the study.

The dose reduction plan for the drugs was as follows: in case of serum creatinine peak above 2.0 mg/dl, cisplatin or ifosfamide has to be reduced to 50% of initial dosage. If serum creatinine on day 1 of new course was >1.5 mg/dl, cisplatin was omitted and ifosfamide dosage reduced by 50%. If the granulocytes or platelets nadir was, respectively, less than 500/mm^3^ or 25,000/mm^3^, drugs were to be given at 75% of the initial dosage. In case of any new cardiac problem, epirubicin was stopped. Failure to recover from myelosuppression (neutrophils < 1,500/mm^3^ or platelets < 100,000/mm3) by day 36 was reason for discontinuation of treatment There was no upward dose modification.

### Workup

The initial workup consisted of a complete history and physical examination, chest X-ray and computed tomography (CT) scan, fiberoptic bronchoscopy with biopsy, bone scintigraphy with X-rays of suspected areas, bone marrow biopsy, liver and adrenals CT scan or echography, brain nuclear magnetic resonance or CT scan, blood chemistries including complete blood cell counts, electrolytes, serum creatinine and liver function tests, ECG, and echocardiogram or isotopic left ventricular ejection fraction. Blood chemistries, chest X-ray, and clinical examination were repeated before each course. Restaging, including all tests performed during the initial workup (except for bronchoscopy), was repeated every three courses. After discontinuation of therapy, patients were assessed every 2 months for 6 months and then every 3 months by clinical examination, blood chemistries, and chest X-ray.

### Evaluation Criteria

Patients were considered evaluable for response if they had completed three courses of chemotherapy. Responses were evaluated during regular meetings of the group by at least three independent observers. Complete remission was defined as the disappearance of all signs of disease for at least 4 weeks. In measurable disease, partial response (PR) consisted of a 50% or greater decrease of the sum of the products of the two greatest diameters of all measurable lesions as established by two observations not less than 4 weeks apart and without the appearance of new lesions or progression of any lesion. Patients with unidimensionally measurable lesions were considered to have evaluable disease. In evaluable disease, PR was defined as an estimated decrease in tumor size of 50%. Progression was defined as an increase of >25% in one or more measurable or evaluable lesions or the appearance of new lesions. All other circumstances were classified as “no change”. Patients with early death due to progression of the disease before any evaluation, those with early disease progression before evaluation, and those with toxic death or early treatment discontinuation due to chemotherapy were considered evaluable.

Progression-free survival (PFS) and survival times were calculated from the day of randomization until the first event, progression, or death for the first item and death for the second item. World Health Organization criteria were used to assess toxicity.

### Statistics

Randomization was stratified by center, Karnofsky PS (≤70 versus ≥80), presence of metastatic disease or not, and neutrophil count (≤ versus >7,500/mm^3^). The procedure was centralized and computerized. Randomization algorithm used the minimization technique (7). Treatment assignment was obtained by calling the study data manager. The ELCWP central office for the study coordination and analysis (including the study coordinator, the biostatistician and the data manager) was located at the Jules Bordet Institute in Brussels.

The primary end point of the trial was survival. On the basis of the systematic review performed by the Group (3), regimens based on cisplatin and etoposide could improve patients’ survival compared to regimens including etoposide but not cisplatin. The benefit in favor of the two drugs was expressed in terms of an HR that was estimated to be 0.70. Our aim was to have a clinical trial able to detect that the HR of death between two such regimens was different from 1 in case its true value is 0.70 (for a patient treated with both drugs compared to a patient receiving a regimen based on etoposide only). Fixing the HR is enough to calculate the number of events required to have the planned statistical power. So, we did not search to translate the HR into an absolute difference. However, assuming a median survival of 10 months in the IVE regimen, an HR of 0.70 in favor of CE translates into a median survival of about 14 months. Such an HR should be detectable with a power of 80% using a test level of 5%. To achieve this objective, we needed to observe an overall number of events (deaths) of 315. Assuming that 90% of the patients will be followed until death, this requirement was equivalent to the randomization of 175 patients per arm in the study. Secondary end points were response and toxicity. No interim analysis for survival or response was done.

Survival curves were estimated by the method of Kaplan and Meier. The log rank test was used to compare survival curves. *p*-Values for testing differences between proportions were calculated with chi-square tests or with Fisher’s exact tests. A multivariate analysis for adjustment of the treatment effect taking into account prognostic factors was carried out for survival by fitting the data with Cox models. Statistical results were considered as significant when the *p* value was <0.05. All reported *p* values are two-sided.

The evaluation of chemotherapy intensity was performed by the calculation of two dose-related variables. The relative dose-intensity (RDI) was defined, for each drug, by the ratio of the received dose divided by the scheduled dose to the actual duration of treatment divided by the scheduled duration. The total RDI was the mean of the RDIs of all the drugs. The RDI was expressed as the percentage of the projected intensity. The absolute dose-intensity expressed in mg/m^2^/3 weeks was defined as the ratio of the received dose to the actual duration of treatment; it was analyzed for etoposide. All of the formulae have been published in a prior report (8). Using Mann–Whitney tests, comparisons of the distributions of the dose intensity variables between regimens were done.

## Results

**Figure 1:**
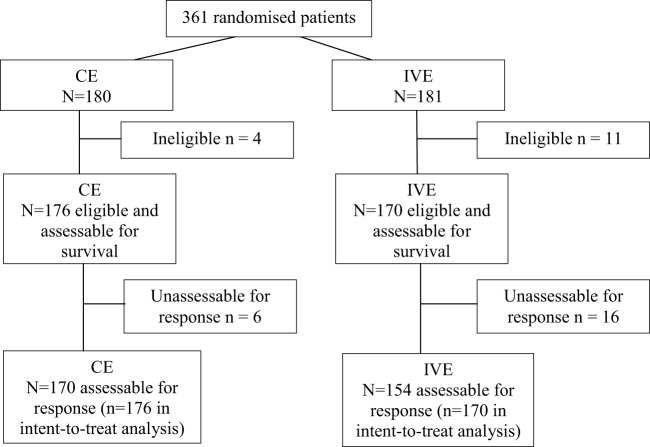
Consort diagram.

A total of 361 patients were randomized from May 2000 to May 2013. Fifteen were not eligible, 4 in the CE arm (2 by history of another cancer and 2 by lack of data), and 11 in the IVE arm (2 by history of another cancer, 2 by lack of data, 3 by wrong histology, 1 because of LD, 1 by increased bilirubinemia, 1 by incomplete initial workup, and 1 by major protocol violation). Of the 346 eligible patients, 176 were allocated to the CE arm and 170 eligible to the IVE arm (Figure 1, CONSORT diagram). Their characteristics are shown in Table 1. Both arms were well balanced. Median follow-up duration of the patients at the time of analysis was 113 months (range: 4–169). There were 329 deaths. Among the 17 patients alive, 9 are lost to follow-up.

**Table 1:** Characteristics of the eligible patients.

Characteristics	CE (*n* = 176)	IVE (*n* = 170)
Age median (min–max)	60 years (40–78)	61 years (42–83)
**Sex**		
Male Female	145 (82%)	129 (76%)
	31 (18%)	41 (24%)
**Karnofsky**		
≤70 ≥80	39 (22%)	41 (24%)
	137 (78%)	129 (76%)
**Disease extent**		
Locoregional Metastatic Unknown	14 (8%)	16 (9%)
	162 (92%)	153 (90%)
	–	1 (1%)
**Weight loss**		
≤5% >5% Unknown	97 (55%)	88 (52%)
	50 (28%)	52 (31%)
	29 (16%)	30 (18%)
**Neutrophil count**		
≤7,500/mm^3^ • >7,500/mm^3^ • Unknown	108 (61%)	97 (57%)
	46 (26%)	56 (33%)
	22 (12%)	17 (10%)

*CE, cisplatin + etoposide regimen*.

Forty-seven percentage of the patients in each arm received six cycles of chemotherapy; 8 and 10% receiving more cycles (up to 9) in the CE and IVE arms, respectively (*p* = 0.66). Dose reductions involved 43 and 54% of the patients, respectively (*p* = 0.07). In terms of dose-intensity, there was no significant difference for RDI with respective median values of 0.80 and 0.76 for the whole chemotherapy (*p* = 0.13) and of 0.79 and 0.77 for etoposide (*p* = 0.34). The median weekly dose of etoposide received was 79 and 77 mg/m^2^/week for the CE and IVE arms, respectively (*p* = 0.34).

Twenty-two patients were not assessable for response: 6 in the CE arm (no treatment given in 2, 1 lost to follow-up, 1 sudden death, 1 no workup for evaluation, 1 treatment stop due to tuberculosis), and 16 in the IVE arm (6 no treatment given, 4 major protocol violation, 3 early death unrelated to cancer or treatment, 1 lost to follow-up, 1 workup refusal, and 1 sudden death). Table 2 shows the evaluation of response to chemotherapy. Two additional responses (one in each arm) were observed after the first three cycles of chemotherapy later. Best objective responses rates (intent to treat analysis) were 106 patients/176, 60% (95% CI 53–68%) for CE and 101/170, 59% (95% CI 52–67%) for IVE (*p* = 0.88), respectively.

**Table 2:** Antitumor response assessment.

Response at 3 cycles	CE	IVE
N patients	176	170
Complete response	2 (1%)	2 (1%)
Partial response	103 (59%)	98 (58%)
No change	9 (5%)	12 (7%)
Progression	21 (12%)	19 (11%)
Early death by cancer	5 (3%)	6 (4%)
Early death by toxicity	18 (10%)	10 (6%)
Death due to tumor necrosis	1 (1%)	3 (2%)
Stop for toxicity	10 (6%)	3 (2%)
Stop by patient for toxicity	1 (1%)	1 (1%)
Inevaluable	6 (3%)	16 (9%)

*CE, cisplatin + etoposide regimen; IVE, ifosfamide + etoposide + epirubicin regimen*.

The number of documented progressions was 251; 89 patients died without documentation of progression. In those 89 patients, death was considered cancer-related by the local investigator but no progression was formally documented by radiological investigations in 16 cases; 22 patients died from infection eventually related to neutropenia, 9 died from cardiovascular events, 12 died from early death by cancer or bleeding due to tumoral necrosis, no cause can be documented in 24 patients while the 6 latter died from digestive (*n* = 2), renal (*n* = 2), respiratory (*n* = 1), or neurological (*n* = 1) events. There was no significant difference between arms in terms of documented progression, primary progression, and secondary progression within 3 months after treatment stop or after more than 3 months after treatment stop. Median PFS for CE and IVE was5.1 months (95% CI 4.8–6.2) and 5.3 months (95% CI 4.7–6.2), respectively, with 1-year rates of 10% (95% CI 5–15%) and 7% (95% CI 3–11%) and 2-year rates of 2% (95% CI 0–4%) and 3% (95% CI 0–6%).

**Figure 2:**
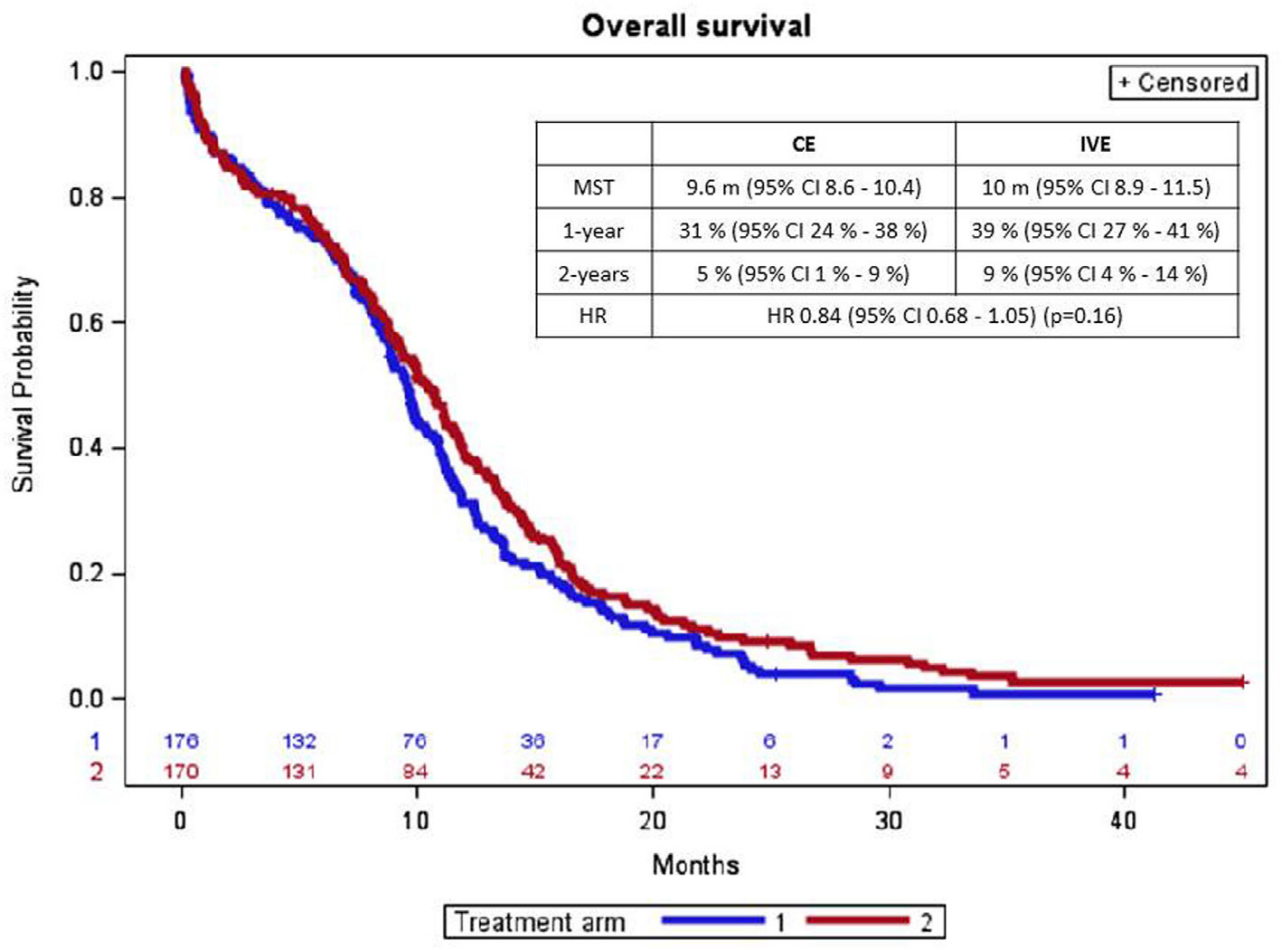
Overall survival curves according to treatment arm (arm 1 = CE; arm 2 = IVE). CE, cisplatin + etoposide regimen; IVE, ifosfamide + etoposide + epirubicin regimen; MST, median survival time; CI, confidence interval; m, month; HR, hazard ratio.

There was no statistically significant difference in overall survival according to treatment arm (Figure 2). 169 and 160 deaths were observed with CE and IVE, respectively. Median overall survival and survival rates at 1 year and at 2 years were (with 95% CI) 9.6 months (8.6–10.4) and 10 months (8.9–11.5), 31% (24–38%) and 39% (27–41%), and 5 (1–9%) and 9% (4–14%) (*p* = 0.16), respectively. HR for treatment arm (taking CE as reference) was 0.84 (0.68–1.05).

The univariate analyses of prognostic factors for survival are detailed in Table 3. Four were significantly associated with survival age (*p* = 0.002), sex (*p* = 0.008), Karnofsky PS (*p* = 0.0001), and neutrophil count (*p* = 0.006). In multivariate analysis, only two were statistically significant: sex with HR = 0.69 (95% CI 0.49–0.97) (*p* = 0.03) and PS with HR = 0.53 (95% CI 0.49–0.97) (*p* < 0.0001). After adjustment for the prognostic factors, HR for treatment arm was 0.83 (95% CI 0.65–1.08), *p* = 0.17.

**Table 3:** Univariate analyses of prognostic factors for survival.

Variable	HR	95% CI	*p*-Value
Age (continuous evaluation)	1.02	1.01–1.04	0.002
Staging (ref = LD)	1.38	0.94–2.04	0.10
Sex (ref = male)	0.69	0.52–0.91	0.008
Karnofsky (ref ≤ 70)	0.60	0.46–0.78	0.0001
Weight loss (ref ≤ 5%)	1.28	1.00–1.64	0.05
Neutrophil count (ref ≤ 75%)	1.42	1.11–1.82	0.006
Neutrophil count (ref ≤ 7,500)	1.46	1.14–1.86	0.003
WBC count (ref = ≤ 100,00)	1.23	0.98–1.54	0.08

*HR, hazard ratio; CI, confidence interval; LD, limited disease*.

Hematological and non-hematological toxicities are described in Tables 4 and 5, respectively. There was more leukopenia with IVE and more thrombopenia and nausea and vomiting with CE. Toxic deaths occurred in 28 patients: 17 in the CE arm and 11 in the IVE arm. Causes were, in the CE arm, complicated febrile neutropenia (8), cardiovascular complications (4), septic shock (2), colic perforation related to steroids administration (1), sudden death (1), and bleeding due to tumor necrosis (1). They were for IVE complicated febrile neutropenia (5), sudden death (2), septic shock (1), respiratory failure (1), renal failure (1), and vascular complication (1).

**Table 4:** Hematological toxicity analysis.

Leukopenia, *p* < 0.0001	Evaluable	0	I	II	III	IV
CE	174	26 (15%)	30 (17%)	45 (26%)	42 (24%)	31 (18%)
IVE	165	22 (13%)	4 (2%)	28 (17%)	58 (35%)	53 (32%)

**Thrombopenia, ***p*** < 0.001**	**Evaluable**	**0**	**I**	**II**	**III**	**IV**

CE	174	86 (49%)	20 (11%)	30 (17%)	23 (13%)	15 (9%)
IVE	165	114 (69%)	16 (10%)	15 (9%)	12 (7%)	8 (5%)

*CE, cisplatin + etoposide regimen; IVE, ifosfamide + etoposide + epirubicin regimen*.

**Table 5:** Non-hematological toxicity analysis.

Toxicity	Evaluable	Grade III/IV	Evaluable	Grade III/IV	*p*
		
	CE		IVE		
Nausea/vomiting	154	17 (11%)	155	8 (5%)	0.06
Diarrhea	153	3 (2%)	155	0	0.08
Skin toxicity	152	–	152	–	–
Infection	156	18 (12%)	155	23 (15%)	0.39
Bleeding	152	2 (1%)	153	1 (1%)	1
Neurological	149	3 (2%)	154	–	0.24
Urinary	152	–	153	–	
Hear loss	152	–	153	–	
Nephrotoxicity	156	3 (2%)	154	2 (1%)	
Stomatitis	151	1 (1%)	155	2 (1%)	
Respiratory	151	3 (2%)	154	10 (6%)	0.10
Cardiac	153	6 (4%)	153	2 (1%)	0.28
Alopecia	151	65 (43%)	152	64 (42%)	0.96

*CE, cisplatin + etoposide regimen; IVE, ifosfamide + etoposide + epirubicin regimen*.

## Discussion

Our randomized clinical trial (RCT) failed to confirm in terms of overall survival the superiority of the CE combination over an etoposide-containing regimen without cisplatin as suggested by our meta-analysis (3). From a clinical point of view, our results do not support clinical practice guidelines recommending platinum–etoposide regimens as the unique chemotherapy reference for first-line treatment of ED SCLC. From a methodological point of view, the study emphasizes the necessity of *ad hoc* controlled trials to validate meta-analysis results before implementation in the daily practice.

The strongest strength of our RCT is its completion with a strict respect of the statistical considerations. We did not perform any preliminary analysis before reaching the number of required events. This explains in part why we had to wait so long before presenting the results in addition to a progressive relative reduction in SCLC epidemiology. Nevertheless, during this time period standard therapeutics for ED SCLC remained globally unchanged in European countries (irinotecan being used in Far East countries) as well as workup procedures. Despite modifications in the TNM classification, our definition of ED is well validated and conserved its operational value at the difference of the TNM staging that mainly has prognostic significance.

Another advantage of the study is to be purely academic and is not disturbed by any commercial considerations. As regimens, we chose those we routinely used in our trials and practice in 1990s. We had nevertheless to face the problem of a slower accrual than expected, precisely due to the academic character of the study. Even if the protocol was approved by the ethical committees and the patients accrual started before the European directive on good clinical practice in the conduct of clinical trials on medicinal products for human use, the burden of its administrative consequence had a psychological effect on the investigators of our Group, some becoming more and more reluctant to propose their patients to participate to therapeutic studies. This negative effect is well discussed in the literature (9), and the directive needs to be improved to facilitate academic research. Nevertheless, today, the conduct of a trial like the presently reported one is very difficult for clinicians because of lack of financial, administrative, and managing resources to face all the bureaucratic constraints resulting from the directive.

Discrepancy between meta-analysis and subsequent RCT is well documented in the literature in various medical disciplines, even when the trials are very large (10–13). Some methodologists recommend large-scale randomized studies to provide adequate evidence for clinical decision, without undue emphasis on small size trials, meta-analysis, and subgroup analysis (14). It can be argued that our study is not large enough. The statistical considerations have been calculated to answer to the question with a sufficient power and a clinical significance. For SCLC, a trial including around 350 patients is not to be considered small, when compared to most recent studies (15) and in the present case, the required number of patients was reached to allow answering the question according to considerations defined prior to starting the enrollment of patients.

Other meta-analyses are available in the literature for first-line chemotherapy in ED SCLC. None deals with our topic. Pujol et al. (16) conducted a meta-analysis of randomized trials of a cisplatin-containing regimen versus a regimen without this alkylating agent. The role of etoposide has not been specifically assessed. The authors concluded to an advantage for using cisplatin-based chemotherapy. Two other groups have compared CE to cisplatin plus irinotecan (17), irinotecan–platinum with etoposide (18, 19) or CE to other platinum-based regimens (20), without showing a superiority of a particular except perhaps cisplatin plus irinotecan, especially in Far East populations. But again, the specific effect of etoposide has not been investigated in this study. The COCIS meta-analysis (21) of individual patient data, comparing carboplatin—or cisplatin—based chemotherapy, did not cover our present topic about the role of etoposide. More recently, a Cochrane meta-analysis (22) looked at the effect of platinum administration versus non-platinum combinations. No improvement in terms of 6, 12, and 24 months survival was noted. Despite the specific questions of adding cisplatin to etoposide versus an etoposide without cisplatin regimen was not addressed, this meta-analysis add to the questioning of the use of platinum derivatives for first-line treatment of ED SCLC.

Excluding alternating regimens, five other randomized trials (23–27) presented with some similarities with our trial. These are summarized in Table 6. Only one included exclusively ED SCLC (24) and for two studies, it was not possible obtaining separate data for ED. Regimens were heterogeneous and in two studies, carboplatin was allowed or the only platinum compound. None of the trials showed improvement in terms of 12- and 24-month survival by adding a platinum derivative to etoposide. The data are also opening the question of the type of platinum derivative to be used in addition to etoposide. Few randomized trials have been published on this topic in SCLC. An individual data meta-analysis published in 2012, including a limited number of patients, showed no difference in terms of overall survival and PFS between carboplatin- and cisplatin-containing regimens while a differential toxicity profile with more hematological toxicities with carboplatin and non-hematological ones for cisplatin (21).

**Table 6:** Summary of randomized trials comparing a platinum–etoposide regimen to an etoposide combination in small-cell lung cancer patients.^a^

Reference	Disease extent	*N*	Schedules	1-year survival	2-year survival
Baka et al. (23)	LD/ED	280 (114 ED)	ACE PE	RR 0.9 (95% CI 0.39–2.10)	RR 4.51 (95% CI 0.22–91.86)
Gatzemeier et al. (24)	ED	317	CEV EV	RR 1.10 (95% CI 0.85–1.43)	RR 1.01 (95% CI 0.60–1.71)
Sculier et al. (25)	LD/ED	201 (102 ED)	PEVs EVs	RR 0.77 (95% CI 0.54–1.08)^b^	RR 0.84 (95% CI 0.37–1.90)^b^
Urban et al. (26)	LD/ED	457 (360)	ACE ACE-P	RR 1.10 (95% CI 0.81–1.50)^b^	RR 1.00 (95% CI 0.44–2.25)^b^
Wolf et al. (27)	LD/ED	141 (87 ED)	PE IE	RR 1.29 (95% CI 0.62–2.69)	RR 0.67 (95% CI 0.16–2.81)

*^a^Adapted from Cochrane 2015 (22)*.

*^b^Results are for combined LD and ED patients*.

*LD, limited disease; ED, extensive or metastatic disease; ACE, adriamycin, cyclophosphamide, etoposide; PE, cisplatin, etoposide; RR, relative risk; CEV, carboplatin, etoposide, vincristine; EV, etoposide, vincristine; PEVs, cisplatin, etoposide, vindesine; EVs, etoposide, vindesine; P, cisplatin; IE, ifosfamide, etoposide*.

Our RCT results have implication for guidelines in the management of SCLC. Today, clinical practice guidelines recommend for ED the administration of a platinum-based chemotherapy with etoposide in America by the American College of Chest Physicians (CHEST) (28, 29) and by the American Society of Clinical Oncology endorsing CHEST (30) and in Europe by the European Society of Medical Oncology (31) endorsed by the Japanese Society of Medical Oncology or by our own group, the ELCWP (15). For LD, the data for supporting the administration of CE are very consistent because almost all the RCTs with radiochemotherapy have used that regimen, mainly for radiosensitization purposes. But for ED, the scientific evidence is supported by the two meta-analyses, by Pujol et al. (16) and by our group (3). All agree that there is no clear survival benefit in favor of platinum-derivative regimens with etoposide. The main argument for this recommendation is a better tolerance. The choice between cisplatin and carboplatin is still open and the lack of benefice when irinotecan is substituted to etoposide is emphasized.

Finally, we can discuss about the optimal duration of chemotherapy in ED SCLC. Numerous trials assessed prolongation of chemotherapy above four to six cycles but with very heterogeneous designs precluding meaningful conclusions. In the present study, 8–10% of the patients received up to nine cycles but the majority had no more than six cycles. We can expect that prolonging chemotherapy duration may expose the patient to more adverse events. As there is no high level evidence on the optimal number of chemotherapy cycle, prolonging treatment above six cycles should be an individual decision taking into account the potential benefit and the risk of toxicity.

In conclusion, according to our meta-analysis (3) but not previous meta-analysis results (3, 16), this original, large, and specifically designed for ED SCLC, randomized, controlled trial showed that the CE combination failed to improve survival in comparison to an etoposide-containing regimen without cisplatin. These data weaken the evidence supporting the currently available guidelines for the management of ED SCLC and may suggest updating them according to our results.

## European Lung Cancer Working Party

Institut Jules Bordet, Brussels, Belgium (J. P. Sculier, T. Berghmans, A. P. Meert, P. Van Houtte, M. Roelandts, M. Paesmans, and N. Leclercq). CHU de Lille, Université de Lille, F59000 Lille, France (J. J. Lafitte and A. Scherpereel). Hospital de Sagunto, Valencia, Spain (V. Giner). Hôpital Civil de Charleroi, Charleroi, Belgium (J. Lecomte and J. Thiriaux). Evangelismos General Hospital, Athens, Greece (C. G Alexopoulos and M. Vaslamatzis). Hôpital Ambroise Paré, Mons, Belgium (S. Holbrechts, P. Wackenier, and P. Recloux). C.H.R. Peltzer-La Tourelle, Verviers, Belgium (Y. Bonduelle and J. L Corhay). Clinique Saint-Luc, Namur, Belgium (O. Van Cutsem and M. Mairesse). Hellenic Cancer Institute, Athens, Greece (A. Efremidis and G. Koumakis). C.H. de Douai, Douai, France (M. C. Florin, E. Maetz, and A. Strecker). Hôpital Warquignies, Boussu, Belgium (M. Richez). CH Tourcoing, Tourcoing, France (X. Ficheroulle). Clinique St-Joseph, Gilly, Belgium (B. Colinet). C.H.I. de Montfermeil, Montfermeil, France (T. Collon and C. Zacharias). C.H. Etterbeek-Ixelles-Moliere, Brussels, Belgium (S. Bensliman). Hôpital Brugmann, Brussels, Belgium (A. Drowart and T. Prigogine). C.H.U. Vésale, Montignies-le-Tilleul, Belgium (D. Brohée). Clinique médico-chirurgicale, Valenciennes, France (B. Stach). RHMS Ath, Belgium (P. Ravez). Hôpital d’Hayange, Hayange, France (M. C. Berchier). Centre Hospitalier du Dr Schaffner, Lens, France (J. Amourette). IMC Mutualité socialiste, Tournai, Belgium (A. Tagnon). C.H. de Roubaix, Roubaix, France (F. Kroll and F. Steenhouwer).

## Ethics Statement

This study was carried out in accordance with the recommendations of World Medical Association Declaration of Helsinki with written informed consent from all subjects. All subjects gave written informed consent in accordance with the Declaration of Helsinki. The protocol was approved by the ethical committees of the participating institutions.

## Author Contributions

TB, AS, A-PM, VG, JL, J-JL, and J-PS are all clinical investigators of the ELCWP, elaborating the protocol and including patients, assessing the data and revising the manuscript. NL is the data manager of the ELCWP and managed the data. MP is the biostatistician of the ELCWP, managed the data, performed the statistical analyses, and revised the manuscript.

## Conflict of Interest Statement

The research was conducted in the absence of any commercial or financial relationships that could be construed as a potential conflict of interest. AS was investigator in France for clinical trials in mesothelioma (Bayer, BMS, Boehringer-Ingelheim, Epizyme, MedImmune/AZ, Morphotek), received financial support for research (Roche, Teva, Pierre Fabre), and was board member (BMS, MSD, Roche).
